# Indel PDB: A database of structural insertions and deletions derived from sequence alignments of closely related proteins

**DOI:** 10.1186/1471-2105-9-293

**Published:** 2008-06-25

**Authors:** Michael Hsing, Artem Cherkasov

**Affiliations:** 1 Bioinformatics Graduate Program, Faculty of Graduate Studies, University of British Columbia, 100-570 West 7th Avenue, Vancouver, BC, V5T 4S6, Canada; 2 Division of Infectious Diseases, Department of Medicine, Faculty of Medicine, University of British Columbia, D 452 HP, VGH, 2733 Heather Street, Vancouver, BC, V5Z 3J5, Canada

## Abstract

**Background:**

Insertions and deletions (indels) represent a common type of sequence variations, which are less studied and pose many important biological questions. Recent research has shown that the presence of sizable indels in protein sequences may be indicative of protein essentiality and their role in protein interaction networks. Examples of utilization of indels for structure-based drug design have also been recently demonstrated. Nonetheless many structural and functional characteristics of indels remain less researched or unknown.

**Description:**

We have created a web-based resource, Indel PDB, representing a structural database of insertions/deletions identified from the sequence alignments of highly similar proteins found in the Protein Data Bank (PDB). Indel PDB utilized large amounts of available structural information to characterize 1-, 2- and 3-dimensional features of indel sites.

Indel PDB contains 117,266 non-redundant indel sites extracted from 11,294 indel-containing proteins. Unlike loop databases, Indel PDB features more indel sequences with secondary structures including alpha-helices and beta-sheets in addition to loops. The insertion fragments have been characterized by their sequences, lengths, locations, secondary structure composition, solvent accessibility, protein domain association and three dimensional structures.

**Conclusion:**

By utilizing the data available in Indel PDB, we have studied and presented here several sequence and structural features of indels. We anticipate that Indel PDB will not only enable future functional studies of indels, but will also assist protein modeling efforts and identification of indel-directed drug binding sites.

## Background

Insertions/deletions (indels) and amino acid substitutions represent the two most common types of sequence variations, observed among similar proteins [1]. Unlike amino acid substitutions, which have been studied intensively in the past years [2,3], indels remain less understood and still pose many biological questions.

Recently, a large-scale indel analysis has been conducted for 136 complete bacterial and protozoan genomes, and the results have shown that up to 5–10% of all proteins contained sizable indels, when compared to human homologues [4]. Our research has further shown possible relationships between indels and protein essentiality [5] and the role of indels in protein-protein interactions. For instance, it has been shown that indel-containing proteins were more likely to be essential than non-indel proteins and involved in more protein-protein interactions [5]. It has also been suggested that sequence insertions and deletions can change protein-protein interactions and modify protein network characteristics [6].

Moreover, it has also been demonstrated that the structural differences of indel sites between pathogen and host proteins can have valuable therapeutic applications, enabling selective targeting of conserved bacterial proteins, but at the same time, eliminating drug cross-reaction with the human homologues [7-9]. For instance, it has been shown that a Leishmania elongation factor contains a 12 amino acid sequence deletion compared with its human homolog, and the deletion site has been used for developing small compounds targeting specifically to the Leishmania protein but not the human protein [9].

Despite the common occurrences of indels and their important roles in protein functions, currently there are no bioinformatics resources that archive structural and sequence information on indel sites derived from sequence alignments of similar proteins. Although early studies have shown us some common features shared by indels in limited datasets [10-15], our understanding of indels can be improved by utilizing the large amount of structural data, as accumulated in Protein Data Bank [16].

Thus we present here, Indel PDB, a structural database of insertion and deletion sites, extracted from aligned protein sequences in PDB. The goal of Indel PDB is to provide a resource of indel 3D structures, which enable various bioinformatics analyses including primary sequence composition, secondary structure assignment, solvent accessibility, length distribution, protein domain association, homology modeling and other comprehensive structural studies. Some of such applications from Indel PDB have been performed and reported in this paper.

Indel PDB is different from loop databases, whose scope is limited to protein loops that lack clear secondary structures [17,18]. For instance in ArchDB [18], which represents one of the most comprehensive loop databases available on the internet, loops are defined as regions that connect the regular secondary structures, extracted from 9587 protein structures. ArchDB classified a total of 58,664 loops (ArchDB95, 13-6-2007) based on their structural similarity with respect to the surrounding secondary structures.

On the other hand, Indel PDB is not limited to loops, but includes all possible gaps (insertions or deletions) present in sequence alignments among closely related proteins in PDB, and therefore such indel sites can possess any possible secondary structures. Although some overlap between Indel PDB and loop databases is expected, Indel PDB features more indel sequences with secondary structures including alpha-helices and beta-sheets in addition to loops. In fact, our analyses have demonstrated that many indels had recognizable 2D structures, in contrast to previous studies that showed most indels had undefined structures and loops [13]. To further distinguish between indels and loops, their differences have been investigated in three aspects: sequence composition, length distribution, and solvent accessibility.

In addition, Indel PDB contains a larger structural database in comparison to ArchDB. Indel PDB is consisted of 117,266 non-redundant indel structures extracted from 11,294 indel-containing proteins. Both the indel structural data and the analysis results are freely accessible through the Indel PDB website [19].

We believe data presented in Indel PDB will not only enable future functional studies of indels, but also facilitate protein modeling of indels and the identification of novel drug binding sites against infectious diseases. Thus, potential users of Indel PDB include 1) molecular biologists who wish to study the functions of particular indel sites by integrating information on protein domains, 2) structural biologists who wish to improve protein homology models or to perform a comprehensive indel structural analysis based on the available indel 3D coordinates, and 3) computational chemists who are searching for potential compound-binding sites of new drug leads by the use of a comprehensive indel search engine available at the Indel PDB website.

## Construction and content

### Construct Indel PDB

**Figure 1:**
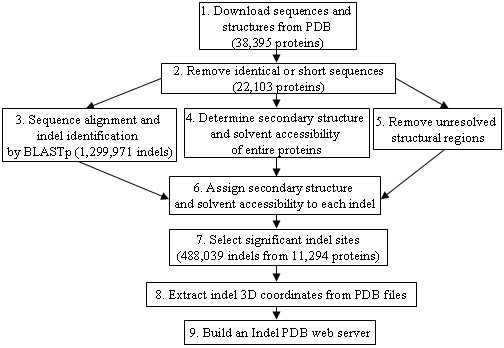
**A flowchart for constructing the Indel PDB web server**. The numbers in brackets indicate proteins or indels remained at each stage of the bioinformatics pipeline.

Building Indel PDB involved nine steps, each of which is described in this section and depicted on Figure 1. In step one, a total of 38,395 PDB structural files and their sequences were downloaded from the PDB website ([20], dated August 30th 2006). In step two, BLASTCLUST (a part of BLAST 2.2.13 package) was used to cluster sequences of the downloaded proteins, based on 100% identity. Proteins that had 100% identical sequences were removed, and only one representative protein was included in the next steps. To avoid short protein sequences, only proteins with 70 or more amino acids were selected for the subsequent parts of the analysis. There were a total of 22,103 proteins that met such criteria.

Furthermore, 22,103 PDB protein sequences were aligned to each other, using BLASTp (version 2.2.13) with the following parameters: e-value ≤ 10^-5 ^and low-complexity filter turned on. The BLAST produced 1,299,971 insertion sites, whose locations and sequences were parsed by in-house Perl scripts, and the results were stored in a MySQL database. Because an insertion site in a query sequence implied a deletion site in a subject sequence, and *vice versa*; only insertions but not deletions needed to be represented in the database. Therefore, in this paper the word 'indel' was used to refer insertion sites on query proteins.

In step four, the DSSP program (obtained from [21] in August, 2006, original paper by [22]) was performed on each of the 22,103 protein structures from step #2 to determine the secondary structures and solvent accessibility. During step 1 to 4, we have noticed there were discrepancies between some 'original' protein sequences as obtained from the PDB website and the 'actual' sequences as stored in the PDB structural files. Such discrepancies were caused by unresolved structural regions or gaps in the crystallographic analysis. Thus, in the fifth step, another BLAST was performed on the original protein sequences against the actual PDB sequences to identify all the unresolved regions in the structural files. This step is important to ensure that the unresolved structural regions in the PDB files were removed from the subsequent indel analyses, so that Indel PDB represents true insertions or deletions in sequence alignments, not the structural gaps in crystallographic analysis. In step number six, the information from step #5 and the original indel positions (step #3) was combined to accurately assign secondary structures and solvent accessibility scores to each of the indel sites.

In the seventh step, indels were selected to be included in the Indel PDB database, based on the following criteria which ensured that the indels were the results of significant BLAST alignments. The BLAST alignment criteria were e-value ≤ 10^-5^, sequence similarity ≥ 50%, and alignment coverage ≥ 80%. In addition, any indel site that contained unresolved region in the PDB structure or low-complexity residues marked by BLAST, was removed from the analysis and excluded from Indel PDB.

In the eighth step, Perl scripts were utilized to extract 3D coordinates of the selected indel sites from the PDB structural files. The indel structures of the same protein were copied into a single PDB file. A total of 11,294 PDB files were produced, which together contained the 3D structures of 488,039 indel sites.

**Figure 2:**
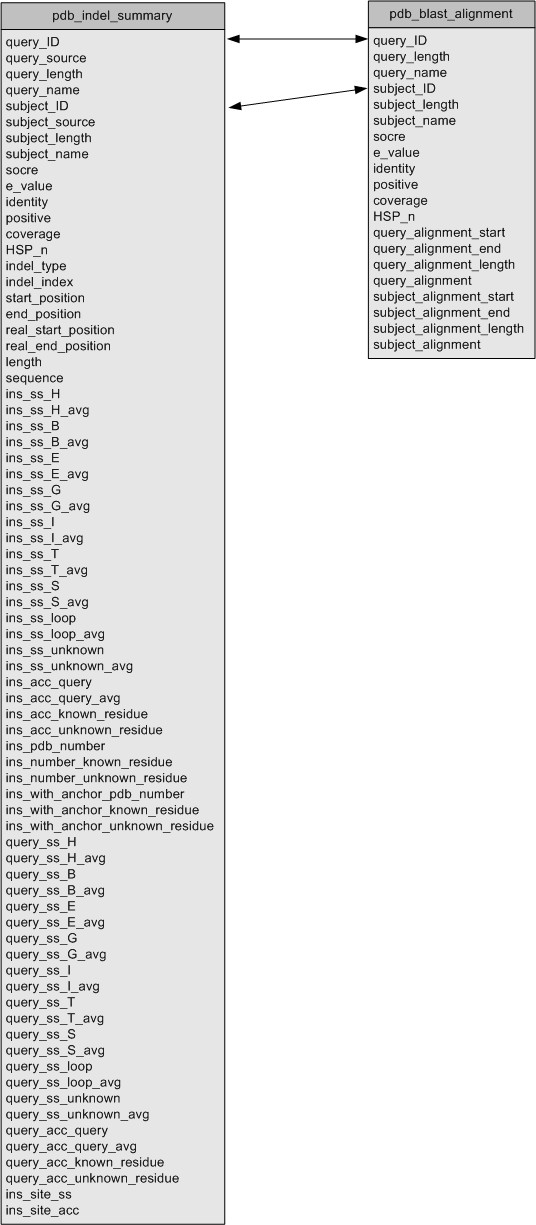
A database schema for Indel PDB.

In the final step, an Apache web server was setup on an IBM Pentium D computer, which links to all the necessary indel information and files stored in a local MySQL database. All of the above indel results are stored in two tables: [indel_pdb_summary] and [pdb_blast_alignment], as shown in Figure 2. The connection between the web server and the MySQL database was established through Perl and CGI.

### Comparative analysis of indels

To demonstrate applications of the Indel PDB database, we utilized the indel data to investigate several indel features that include sequence composition, length distribution, secondary structure composition, and solvent accessibility. All of the analyses operated on a non-redundant set of indel sites, which were extracted from the original set of 488,039 indels by grouping together indel sites with the same start and end position on the same protein. The resulting non-redundant set contains 117,266 indel sites. The values required for each of the analyses were retrieved from the MySQL database using Perl scripts.

The analyses of amino acid sequence and secondary structure composition were repeated on both the indel sites and the full-length indel-containing proteins (referred as indel proteins). Data obtained from indel proteins were treated as background values that were compared to the indel site data. Chi-square test was applied to evaluate if the differences between indel sites and indel proteins were significant. For instance, in the case of comparing the alpha-helix content (H) between indel sites and indel proteins (our samples), the percentages of residues that were H or non-H in both samples were calculated. Then a Chi-square test value was calculated and a P-value was assigned. The same process was repeated for the other secondary structures or the sequence compositions.

Solvent accessibility was measured by (the number of water molecules in contact with a residue) multiplied by 10 or (residue water exposed surface in Angstrom)^2^, according to the DSSP program. Two sample t-test was applied to compare the differences of solvent accessibility between indel sites and indel proteins.

### Length distribution

The indel and loop length distributions were modeled by the Weibull [23] and power law distributions. The Weibull distribution can be described by the function:

S(x) = exp{-(x/α)^β^}, × ≥ 0, α,β > 0

where S(x) is the survival function, and α and β represent a scaling factor and a shape parameter, respectively. The double logarithmic transformation of the Weibull function was performed:

log(-log(S(x)) = β log(x) – β log(α)

The survival function, S(x), is the probability that a variable X has a value greater than a number x. S(x) was calculated by dividing the number of indels with more than x residues by the total number of indels, where x ranges from 1 to 49. If the Weibull distribution can accurately model the indel length distribution, the double logarithmic plot is expected to be linear. The Pearson correlation coefficient (r^2^) as implemented in MS Excel was used to evaluate the linearity of the resulting plot.

The power law distribution is represented by the function:

S(x) = ax^-b^

The logarithmic transformation of the function is:

log(S(x)) = log(a) – blog(x)

Therefore, the fitness of the power law function for the indel/loop length distribution has been evaluated based on the Pearson correlation coefficient (r^2^) of the linear plot.

### Location analysis of protein domain and indel

To further study functional aspects of indels presented in Indel PDB, we have investigated the presence of protein domains that were in the proximity of indel sites. First, 9,318 protein domain profiles characterized by Hidden Markov Model (HMM) were obtained from the Pfam database (version 22.0, [24]). Second, the HMMER program (ver 2.3.2, [25]) was utilized to scan each of the 22,103 PDB protein sequences against each of the 9,318 Pfam domain profiles. The scanning processes were performed on a cluster of 50 CPUs to generate outputs, which contained the exact starting and ending amino acid residues where protein domains were located for each of the protein sequences. In step three, the locations of the protein domains were overlaid with the locations of 117,266 indel sites in 11,294 indel-containing proteins. From an indel perspective, we calculated the distance between any given indel site and all domains on a given protein. The distance was measured by the number of amino acid residues between the boundary of the indel site and a domain site. If there was an overlap between the residues of the indel and the domain, the distance was assigned a "0".

Based on the locations of the indels and the domains, we have computed the overall percentages of indel sites that overlapped with domains, and vise versa, the overall percentages of domain sites that overlapped with indels. In addition, the top 20 protein domains with the highest percentages of overlapping indels were reported with P values determined by Fisher's exact test. The Fisher's exact test was performed with a 2 × 2 contingency table (column 1: indel containing protein, column 2: non-indel containing protein, row 1: contained a domain that overlapped with an indel, row 2: not contained a domain that overlapped with an indel)

## Utility and Discussion

### Overview of Indel PDB

Indel PDB contains sequence and structural data associated with 488,039 (or 117,266 non-redundant) indel sites, extracted from 11,294 indel-containing proteins in PDB. Indel PDB and the indel analysis results are freely accessible to the public over the internet on the World Wide Web [19].

An easy way for users to interact with indel data is through a comprehensive indel search engine. Users can search indels using one or more of the following criteria, including PDB ID, indel length, secondary structure composition, solvent accessibility score, and proximity with protein domains. In addition, users can specify the sources (species) of query and subject proteins. For example, the various searching criteria can be used to identify indels of interests between pathogens and humans for possible drug target binding sites. Furthermore, users can set a specific range on indel length, secondary structure or solvent accessibility to find indel sites that are, for instance, long, mainly alpha-helical, and surface exposed. Moreover, users can search for indels that overlap with certain protein domains by turning on the domain search option, setting the proximity domain distance to '0' and giving a specific domain name or ID (e.g. Peroxidase or PF00141). Such results are useful to study the functional roles of indels among similar proteins.

Alternatively, a query protein sequence can be submitted and searched against all the indel sequences in Indel PDB by BLASTp. Successfully indel hits are displayed to users.

**Figure 3:**
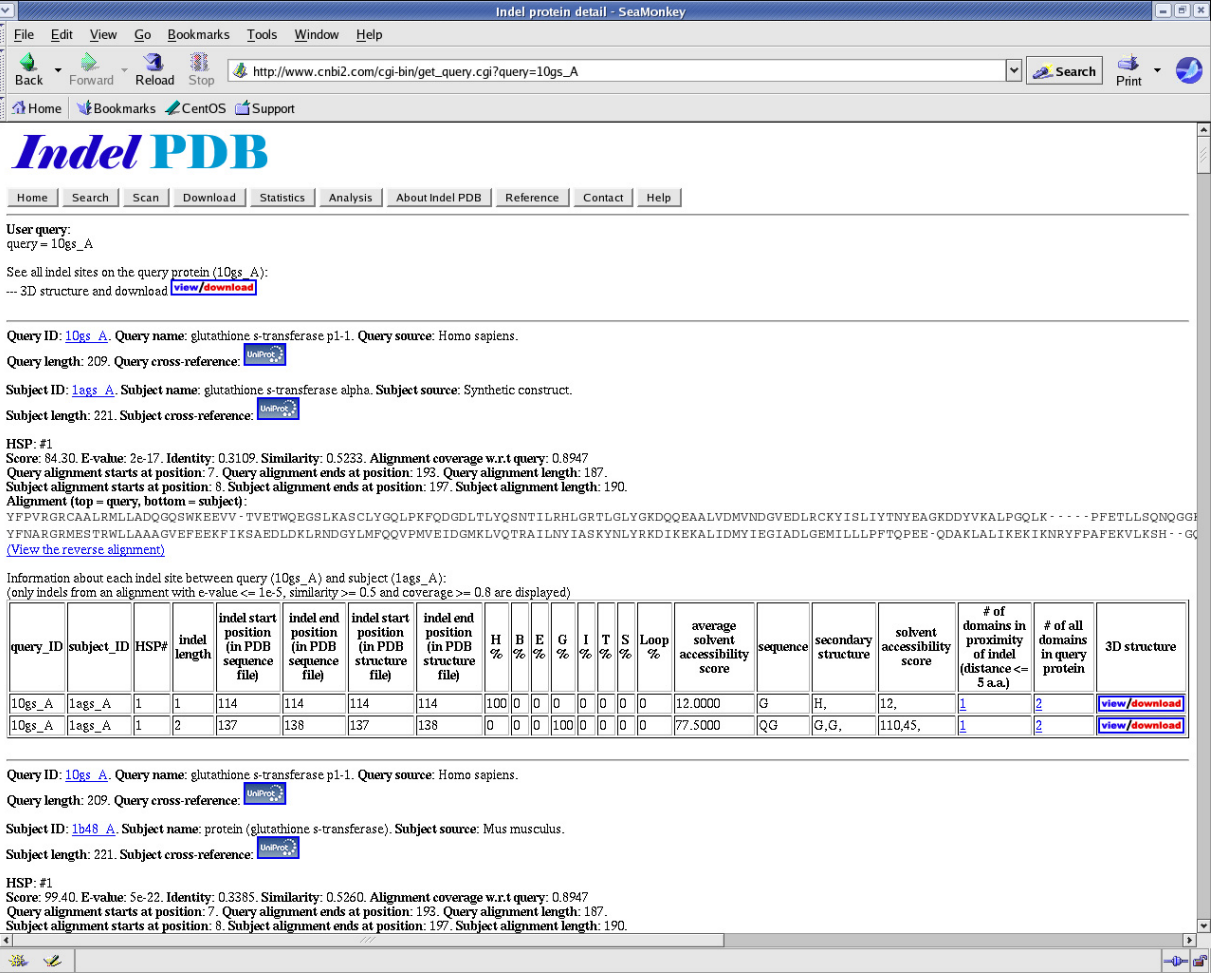
**An indel detail page on Indel PDB**. The navigation buttons on the top of the page provide easy access to different functionality of the website. The indel report on the query protein, 10 gs_A is shown. The screenshot displays indel sites between the query and one of its subject proteins (1ags_A). A detailed BLAST alignment report, followed by an indel summary table is shown.

As shown in Figure 3, the following information of each indel site is displayed: Query PDB ID (protein that contains the insertion site), Query name, Query source, Subject PDB ID (protein that contains the corresponding deletion site), Subject name, Subject source, BLAST alignment scores, the complete sequence alignment, indel location (start and end position on the query protein), indel length, indel sequence, indel secondary structure composition, and indel solvent accessibility scores. Moreover, each indel protein has been cross-referenced to the UniProt database for comprehensive functional annotations. Furthermore, the page shows the number of protein domains that are in proximity of the indel sites, with links to additional information on the domains. The "help" function on each webpage contains more detailed information on web site navigation and display.

**Figure 4:**
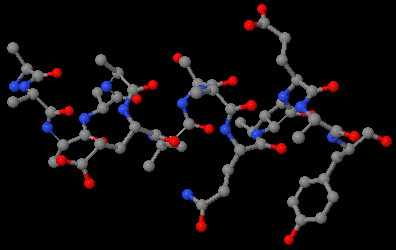
**A 3D view of an indel site by the Jmol applet on Indel PDB**. The indel site (insertion) is 14 amino acid long, present on a query protein, 1EDO_A (Beta-keto acyl carrier protein reductase), in an alignment with a subject protein, 1UZL_A (3-oxoacyl-[acyl-carrier protein] reductase). The indel site has an alpha-helix structure.

In addition, users can visualize each indel 3D structure on Indel PDB by a Jmol JAVA applet [26]. As an example, a 3D view of a 14-amino acid indel site, with an alpha-helix structure, between 1EDO_A and 1UZL_A is shown in Figure 4. Indel PDB includes not only the 3D atomic coordinates of each indel, but also the anchoring residues up to 6 amino acids on each side of the indel, which can be used for protein homology modeling of the indel regions. The indel 3D structure files can be downloaded directly from the Indel PDB website.

In the following sections, we demonstrated the applications of Indel PDB to characterize the structural features of indels. In particular, we studied the sequence composition, length distribution, secondary structure composition, solvent accessibility and domain association of indels in known protein structures. The results obtained are important for understanding the functions of indels and their roles in protein essentiality, protein-protein interactions and drug design. For example, the results on solvent accessibility and secondary structure composition will enable the identification of surface exposed indel sites with unique structural conformation, which can be applied to design novel drug binding site for bacteria and their host proteins. Moreover, each indel site in Indel PDB has a start and end location with respect to its PDB sequence, and thus the indel locations can be mapped to nearby protein domains to investigate the functions of the indels and their potential ligand-binding ability. In addition, the sequence composition results enable studying the bias of amino acid usage on indel sites. Finally, the length distribution models of indels can provide insights about indel abundance among related proteins.

### Sequence composition of indels

**Figure 5:**
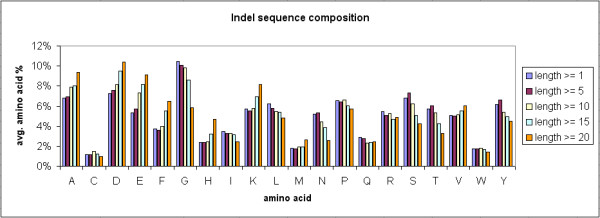
Amino acid composition of indel sequences.

The average sequence composition of the 20 amino acids in each of the 117,266 indel sites was calculated, and the calculation was repeated in the full-length sequences of the11,294 indel-containing proteins, where those indel sites were extracted from. Indels and their indel proteins were classified into four groups according to their length: indels with ≥ 1, ≥ 5, ≥ 10, ≥ 15, or ≥ 20 residues. Additional file 1 summarizes the sequence composition results and the corresponding chi-square and p values of each indel length category, by comparing indel sites and their indel-containing proteins. The average amino acid composition of indels with different minimal length is depicted in Figure 5. As shown in the Figure, the average residue percentages of A, D, and E increased, but those of G, L, N, S, T, and Y decreased when the length of indels went up. The rest of amino acids did not show any clear trend among different indel-length groups. This result indicated that residues such as Alanine (A) and Glutamic acid (E), which prefer a helix conformation [27], are more frequently observed as the length of indels increased. This observation is supported by the later analysis of secondary structural composition, which showed an increase of alpha-helix content (H) in indels as length increased.

**Figure 6:**
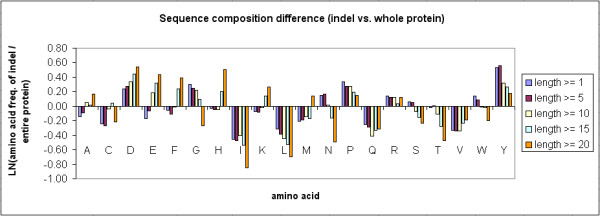
**The difference of average amino acid composition between indel sites and full-length protein sequences**. The y-axis shows the natural logarithm of the ratio of average amino acid frequency in the indel sites to that in the full-length protein sequences.

Figure 6 shows the natural logarithm of the ratio of average amino acid frequency in the indel sites to that in the full-length protein sequences. Some trends can be easily identified from the Figure. For instance, indel sites contained more D, P, and Y in comparison to the entire protein sequences, while I, L, Q, T and V were reduced in indel sites. The differences are significant at P value < 0.001, based on the chi-square tests.

**Figure 7:**
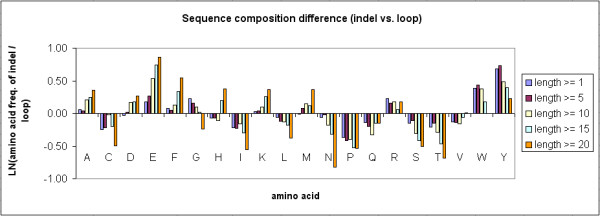
**The difference of average amino acid composition between indel sites and loop sequences**. The y-axis shows the natural logarithm of the ratio of average amino acid frequency in the indel sites to that in the loop sequences.

To compare the sequence composition of indels to that of loops (protein regions that lack any defined secondary structures), the average amino acid frequency of all loops in the 11,294 indel-containing proteins has been computed. A total of 310,103 of loops of various lengths have been identified from the proteins. As shown in Figure 7, the indel sites contained more A, D, E, F, K, M, R, W, Y residues in comparison to the loops, but less C, I, L, N, P, Q, S, T, V residues. The differences are significant at P value < 0.001, based on the chi-square tests. Additional file 2 summarizes the sequence composition results and the corresponding chi-square and p values of each indel length category, by comparing indel sites and loops.

### Length distribution of indels

**Figure 8:**
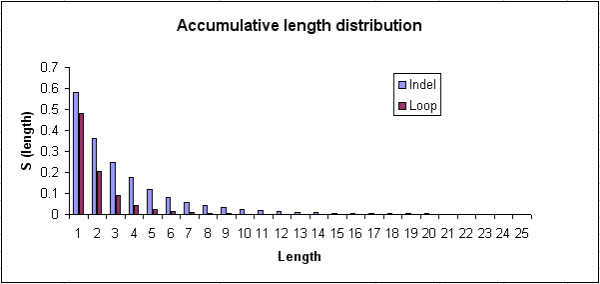
**Accumulative indel length distribution**. The survival function of indel and loop lengths were plotted against a range of lengths from 1 ~25 residues.

Our previous indel studies have shown that indel length distribution could be accurately modeled by the Weibull distribution [4,5]. Therefore, in the current study the Weibull distribution was used to model the length distribution of the 117,266 indel sites and 310,103 loops from Indel PDB. In addition to the Weibull function, the length distributions of indels and loops have been fitted to a power-law function. The survival function over a range of indel or loop lengths (from 1–25) was plotted on Figure 8, indicating that there were many short indels/loops but very few longer indels/loops. The number of indels and loops both reduced as the length increased, however, the number of loops reduced as a faster rate than indels. Moreover, the maximal loop length was 26 amino-acid long, while the maximal indel length was 50 in the Indel PDB dataset.

**Figure 9:**
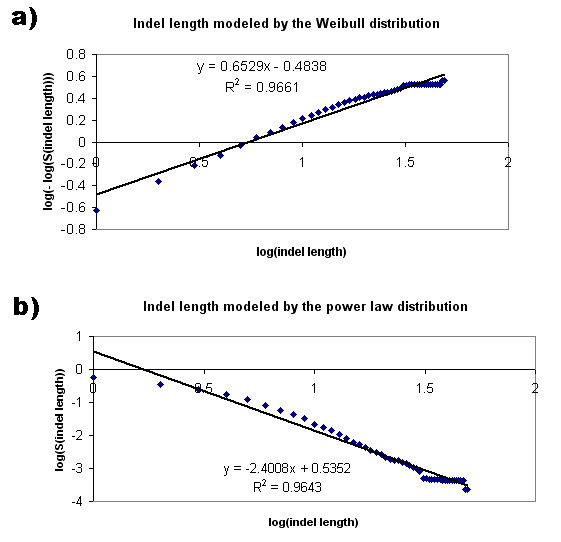
**Indel length modeled by the Weibull and power law distribution**. a) The double logarithm of the survival function in Weibull distribution was plotted against the logarithm of indel length, which ranged from 1 to 49 residues. The plot was fitted onto a liner line with R^2 ^= 0.9661, indicating a good fit of the Weibull distribution. b) The logarithm of the survival function in power law distribution was plotted against the logarithm of indel length, which ranged from 1 to 49 residues. The plot was fitted onto a liner line with R^2 ^= 0.9643.

**Figure 10:**
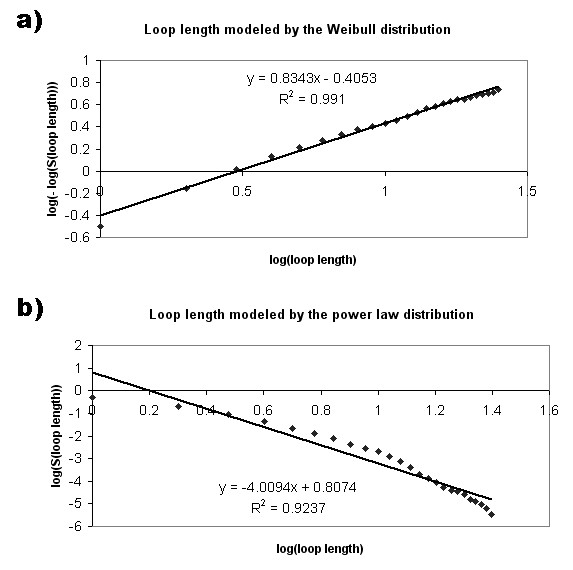
**Loop length modeled by the Weibull and power law distribution**. a) The double logarithm of the survival function in Weibull distribution was plotted against the logarithm of loop length, which ranged from 1 to 25 residues. The plot was fitted onto a liner line with R^2 ^= 0.991, indicating a good fit of the Weibull distribution. b) The logarithm of the survival function in power law distribution was plotted against the logarithm of loop length, which ranged from 1 to 25 residues. The plot was fitted onto a liner line with R^2 ^= 0.9237.

Figure 9a shows a double logarithmic plot of the survival function versus the logarithm of the indel length. The plot could be fitted onto a liner line with R^2 ^value of 0.9661, indicating a good fit of indel length distribution by the Weibull distribution. In Figure 9b, the indel length distribution was fitted into a logarithmic transformation of a power law function, with R^2 ^value of 0.9643. The loop length distribution has been fitted to the Weibull function (Figure 10a) with R^2 ^value of 0.991, and the power law function with R^2 ^value of 0.9237 (Figure 10b).

Thus, the Weibull function has a better fit of the length distribution of indels and loops, in comparison to the power law function. In addition, the results suggest that the occurrence of indels in the studied PDB proteins cannot be attributed to random processes (when normal distribution behaviors would be expected), and the indel lengths are likely to be associated with certain evolutionary mechanisms.

### Secondary structure composition of indels

**Figure 11:**
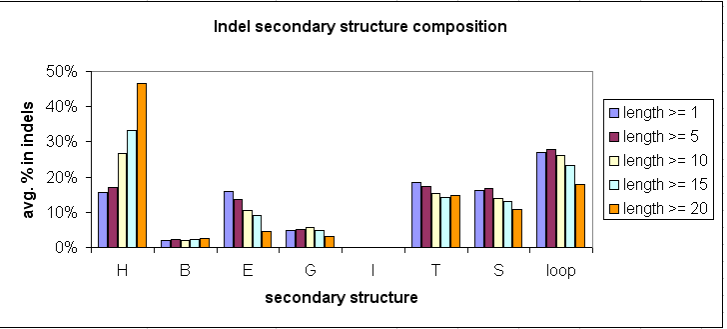
**Average secondary structure composition of indels**. Secondary structures were defined by DSSP (Kabsch and Sander 1983): H = alpha-helix, B = residue in isolated beta-bridge. E = extended strand, participates in beta-ladder, G = 3-helix, I = 5-helix, T = H-bonded turn, S = bend, and loop = undefined structure.

**Figure 12:**
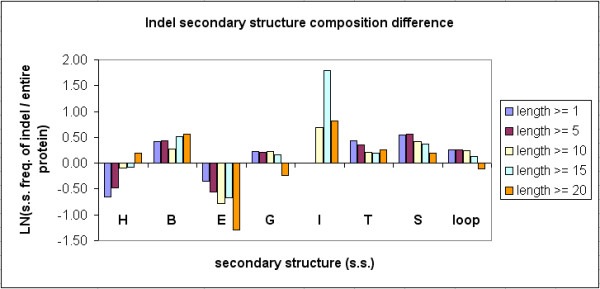
**Difference of average secondary structure composition between indel sites and full-length protein sequences**. The y-axis shows the natural logarithm of the ratio of average secondary structure frequency in the indel sites to that in the full-length protein sequences.

We have assigned secondary structures to each of the 11,294 indel-containing proteins and their 117,266 indel sites in Indel PDB. Secondary structures were defined and assigned by DSSP [22] and its computer program [21]. Additional file 3 summarizes the secondary structure composition results and the corresponding chi-square and p values of each indel length category, by comparing indel sites and their indel-containing proteins. Figure 11 illustrates that when the indel length increased, there was an increase of alpha-helix content (H) in indels, while the percentages of extended beta-strands (E), H-bonded turns (T), bends (S) and loops decreased. In comparison to the indel proteins (as shown in Figure 12), indel sites have increased percentages of T, S, and loop structures, but reduced contents of alpha helices and beta strands. The differences are significant at P value < 0.001, based on the chi-square tests. The proportion of alpha-helices in shorter indels was lower, comparing to the indel protein sequences. However, longer indels had comparable or higher H content than the indel proteins.

In contrast to previous findings [12,13], our results showed that many indel sites had recognizable secondary structures such as alpha helices and beta sheets, in addition to loops or turns.

### Solvent accessibility of indels

The DSSP program was utilized to predict solvent accessibility of the indel proteins and their indel sites. Table 1 indicates that indel sites in all five length groups had higher average of solvent accessibility values than the indel proteins. The differences of solvent accessibility values were significant at P value of 0. The result showed that indel sites were more exposed to the protein surface than average residues of the proteins.

**Table 1:** Solvent accessibility of indel sites and indel proteins.

**Minimal indel length**	1	5	10	15	20
**Indel – avg. solvent accessibility**	59.53	60.36	60.03	58.46	60.50
**indel protein – avg. solvent accessibility**	44.68	43.52	43.46	42.05	42.80
**T test value**	168.20	130.52	73.96	45.96	34.83
**P value**	0	0	0	0	0

Solvent accessibility was measured by (the number of water molecules in contact with a residue) multiplied by 10 or (residue water exposed surface in Angstrom)^2^, according to the DSSP program. The higher the solvent accessibility value, the more exposed the residue is.

In addition, the solvent accessibility of indels has been compared to that of loops. Table 2 indicates that indel sites in all five length groups had higher average of solvent accessibility values than the loops with a significant P value of 0. The result showed that indel sites were more exposed to the protein surface than the loops.

**Table 2:** Solvent accessibility of indel sites and loops.

**Minimal indel length**	1	5	10	15	20
**indel – avg. solvent accessibility**	59.53	60.36	60.03	58.46	60.50
**loop – avg. solvent accessibility**	49.26	47.62	47.55	44.64	44.21
**t test value**	98.74	85.06	47.53	32.38	25.87
**p value**	0	0	0	0	0

Solvent accessibility was measured by (the number of water molecules in contact with a residue) multiplied by 10 or (residue water exposed surface in Angstrom)^2^, according to the DSSP program. The higher the solvent accessibility value, the more exposed the residue is.

### Proximity of indels and protein domains

Previous studies have suggested the roles of indels in modification of protein functions and interactions. Thus, it is possible to anticipate that such indel-directed modifications may occur in the proximity of protein functional or structural domains. To determine the percentage of indel sites that overlapped with at least one protein domain, we have calculated the relative distance between each indel site and a domain on all 22,103 PDB proteins. The average length of domains was 151.4 amino-acid long, with a minimal length of 8 and a maximal length of 1289. As shown in Table 3, among domains of all possible lengths, 93.66% of all indel sites overlapped with at least one domain. Among domains that were equal or less than the average length, 47.33% of all indel sites overlapped with at least one domain.

**Table 3:** Percentage of indel sites that overlapped with at least one protein domain.

**Domain length**	**# of indels overlapping with a domain**	**%**	**# of indels not overlapping with a domain**	**%**	**Total # of indels**
< = 1289	109835	93.66%	7431	6.34%	117266
< = 152	55503	47.33%	61763	52.67%	117266

From a domain perspective, a total of 31,700 instances of domains have been found on the 22,103 PDB proteins. Table 4 indicates that among domains of all possible lengths, 45.22% of the domains overlapped with at least one indel site, and among domains that were equal or less than the average length, 25.94% of the domains overlapped with an indel.

**Table 4:** Percentage of domains that overlapped with at least one indel site.

**Domain length**	**# of domains overlapping with a indel**	**%**	**# of domains not overlapping with a indel**	**%**	**Total # of domains**
< = 1289	14336	45.22%	17364	54.78%	31700
< = 152	8222	25.94%	23478	74.06%	31700

In addition, for each indel-overlapping domain, we have calculated the faction of the number of proteins with such a domain overlapped with an indel to the total number of proteins where the domain was present. Table 5 shows the top 20 over-represented indel-overlapping domains with P-values determined by the Fisher's exact test. Several enzymatic domains such as peroxidase, nitric oxide synthase, and catalase are among the top 20 list, and therefore the result has suggested some possible functional roles of indels, participating in the modification of enzymatic activity of those proteins.

**Table 5:** Top 20 domains that overlapped with indels.

**Domain ID (Pfam)**	**Domain Name**	**# of proteins with the domain overlapping with an indel**	**Total # of proteins with the domain**	**Domain fraction**	**P-value (one tail) from Fisher's exact test**
PF00141	Peroxidase	88	88	1	1.85E-26
PF00565	Staphylococcal nuclease homologue	44	44	1	1.42E-13
PF02876	Staphylococcal/Streptococcal toxin, beta-grasp domain	33	33	1	2.33E-10
PF02898	Nitric oxide synthase, oxygenase domain	31	31	1	8.94E-10
PF00199	Catalase	30	30	1	1.75E-09
PF00232	Glycosyl hydrolase family 1	30	30	1	1.75E-09
PF00502	Phycobilisome protein	29	29	1	3.43E-09
PF01327	Polypeptide deformylase	24	24	1	9.92E-08
PF00896	Phosphorylase family 2	23	23	1	1.94E-07
PF00490	Delta-aminolevulinic acid dehydratase	22	22	1	3.81E-07
PF01742	Clostridial neurotoxin zinc protease	21	21	1	7.45E-07
PF00022	Actin	20	20	1	1.46E-06
PF00274	Fructose-bisphosphate aldolase class-I	20	20	1	1.46E-06
PF00503	G-protein alpha subunit	20	20	1	1.46E-06
PF00224	Pyruvate kinase, barrel domain	17	17	1	1.10E-05
PF03414	Glycosyltransferase family 6	16	16	1	2.15E-05
PF00217	ATP:guanido phosphotransferase, C-terminal catalytic domain	15	15	1	4.21E-05
PF00343	Carbohydrate phosphorylase	15	15	1	4.21E-05
PF00113	Enolase, C-terminal TIM barrel domain	14	14	1	8.24E-05
PF00162	Phosphoglycerate kinase	13	13	1	1.61E-04

Overall the results have indicated that a great number of indel sites overlapped with the locations of protein domains, and therefore it is possible to hypothesize that some of such indel sites are associated with the change of protein functions through domain modifications in evolution.

## Conclusion

We presented Indel PDB, a free web-based resource that contains information on structural insertions and deletions in proteins that have been derived from alignments of closely related sequence. The developed Indel PDB resource aims to facilitate bioinformatics analysis of 1-, 2- and 3-dimensional features of indel sites and their roles in protein essentiality, protein-protein interactions, homology modeling and drug design.

The analysis of the database content demonstrated that indel sites had certain bias of amino acid usage and that indel tended to occur on solvent exposed areas of proteins. In addition, it has been shown that protein indels possessed distinguishable secondary structure composition where loops, turns and bends were the most abundant structural features followed by alpha-helix and beta-sheet containing fragments. It has also been demonstrated that indel length distribution could be accurately described by Weibull function. Moreover, a great number of indel sites have overlapped with locations of protein domains, and the result suggests a possible association between indel occurrences and modifications of protein function.

We anticipate that further applications of Indel PDB in conjunction of protein domain and drug databases will enable identification of novel indel-based drug binding sites for computer-aided drug discovery.

## Availability and requirements

Indel PDB is freely available over the internet on the World Wide Web [19].

## Authors' contributions

MH acquired and analyzed protein sequence and structural data from PDB, designed and implemented the Indel PDB database and website, carried out the indel analyses, and drafted and revised the manuscript. AC conceived and designed the study, and revised the manuscript.

## Supplementary Material

Additional File 1 Table S1. Click here for file

Additional File 2 Table S2. Click here for file

Additional File 3 Table S3. Click here for file
